# Reproducible Lipid Alterations in Patient-Derived Breast Cancer Xenograft FFPE Tissue Identified with MALDI MSI for Pre-Clinical and Clinical Application

**DOI:** 10.3390/metabo11090577

**Published:** 2021-08-26

**Authors:** Vanna Denti, Maria K. Andersen, Andrew Smith, Anna Mary Bofin, Anna Nordborg, Fulvio Magni, Siver Andreas Moestue, Marco Giampà

**Affiliations:** 1 Proteomics and Metabolomics Unit, Department of Medicine and Surgery, University of Milano-Bicocca, 20854 Vedano al Lambro, MB, Italy; v.denti@campus.unimib.it (V.D.); andrew.smith@unimib.it (A.S.); fulvio.magni@unimib.it (F.M.); 2 Department of Circulation and Medical Imaging, NTNU–Norwegian University of Science and Technology, 7491 Trondheim, Norway; maria.k.andersen@ntnu.no; 3 Department of Clinical and Molecular Medicine, NTNU–Norwegian University of Science and Technology, 7491 Trondheim, Norway; anna.bofin@ntnu.no (A.M.B.); siver.a.moestue@ntnu.no (S.A.M.); 4 Department of Biotechnology and Nanomedicine, SINTEF, 7034 Trondheim, Norway; anna.nordborg@sintef.no; 5 Department of Pharmacy, Nord University, 8026 Bodø, Norway

**Keywords:** MALDI MSI, lipidomics, breast cancer, FFPE tissue, diagnosis

## Abstract

The association between lipid metabolism and long-term outcomes is relevant for tumor diagnosis and therapy. Archival material such as formalin-fixed and paraffin embedded (FFPE) tissues is a highly valuable resource for this aim as it is linked to long-term clinical follow-up. Therefore, there is a need to develop robust methodologies able to detect lipids in FFPE material and correlate them with clinical outcomes. In this work, lipidic alterations were investigated in patient-derived xenograft of breast cancer by using a matrix-assisted laser desorption ionization mass spectrometry (MALDI MSI) based workflow that included antigen retrieval as a sample preparation step. We evaluated technical reproducibility, spatial metabolic differentiation within tissue compartments, and treatment response induced by a glutaminase inhibitor (CB-839). This protocol shows a good inter-day robustness (CV = 26 ± 12%). Several lipids could reliably distinguish necrotic and tumor regions across the technical replicates. Moreover, this protocol identified distinct alterations in the tissue lipidome of xenograft treated with glutaminase inhibitors. In conclusion, lipidic alterations in FFPE tissue of breast cancer xenograft observed in this study are a step-forward to a robust and reproducible MALDI-MSI based workflow for pre-clinical and clinical applications.

## 1. Introduction

Metabolic reprogramming is a hallmark of cancer, and can be exploited for both diagnostic and therapeutic purposes [1]. Lipid metabolism is particularly found to be altered in many cancer types including breast cancer [2]. The elevated levels of phospholipids and their metabolic building blocks are believed to support the continuous membrane synthesis during cancer cell growth [3]. Using various analytical techniques, rigorous studies of metabolic networks in cancer have improved our understanding of the disease and helped identify prognostic and predictive biomarkers [4]. It is now widely recognized that metabolism plays an integrated role in cancer cell signaling, and that it represents an important interface between cancer cells and the physical and biological microenvironment of tumors [5].

Understanding the metabolic abnormalities in tumors and the metabolic dependencies between cancer cells and other compartments of a tumor requires analytical tools that are not only sensitive, precise, and accurate, but also allow for the assessment of the metabolic heterogeneity of tumor tissue. Current analytical techniques such as liquid and gas chromatography mass spectrometry (LC-MS and GC-MS) are highly sensitive, but depend on homogenization and extraction of tissue and are therefore unable to provide spatially resolved metabolic information [6,7].

Mass spectrometry imaging (MSI) represents a paradigm shift in cancer metabolomics as it allows for the determination of a multitude of chemical species with high spatial resolution, accompanied by histopathological analysis of the specimen for co-registration of metabolic and biological characteristics [8]. However, while most MSI modalities are suited for the analysis of fresh frozen tissue specimens, most clinical biopsies are stored for years as formalin-fixed paraffin-embedded (FFPE) tissue. The long-term storage makes the FFPE tissues valuable archival material for investigating associations between clinical history and disease. The study of FFPE cohorts permits correlation between biological characteristics and patient survival rate, contributing to the understanding of cancer heterogeneity [9]. For translational research aiming to understand the association between the metabolic state of tumors and the long-term outcome of human disease, there is a need to develop MSI protocols that can determine the levels of low-mass metabolites, lipids, and proteins in a robust and reliable manner.

Matrix-assisted laser desorption and ionization (MALDI) MSI can be used to determine both low-mass molecules and proteins in FFPE tissue [10,11]. However, analysis of lipids has proven difficult in this type of tissue due to their depletion during initial processing with organic solvents, which is further compounded by the remaining lipids being implicated in cross-binding with other structures in the tissue [12,13,14]. For this reason, protocols that are able to remove, or reverse, the cross-links and liberate these trapped biomolecules are currently being developed [15]. Among these, it was recently shown that the inclusion of an antigen retrieval step in the MALDI-MSI sample preparation protocol increased the number of certain solvent-resistant lipids, particularly phosphatidylcholine (PC), sphingomyelin (SM), and phosphatidylserine (PS) species, that can be detected [16]. Moreover, these remaining lipids also maintain some of the biological information present within the different histopathological features of the tissue, being able to distinguish among normal, tumor, and necrotic regions in a small cohort of renal cancer tissue. Thus, mapping the tissue lipidome in FFPE specimens may still provide relevant molecular information that can be exploited when studying the metabolic heterogeneity of tumors. In this work, we have evaluated the robustness of the antigen retrieval MALDI-MSI approach for analysis of lipids in FFPE xenograft breast tumor tissue. Furthermore, we also aimed to determine whether it was possible to identify metabolic responses to the glutaminase inhibition based on alterations in lipid content.

## 2. Results

### 2.1. Evaluation of Technical Reproducibility in Patient Derived Breast Cancer Xenograft

A master peak list was created and contained 26 *m*/*z* values that were identified as reproducible. This peak list contained masses that were reproducibly detected in tumor or necrosis (CV of peak intensity < 45% across technical replicate), and/or had a reproducibly intensity ratio between the necrotic and tumor region (CV of log_2_FC < 60%). Table 1 presents the *m*/*z* values, the coefficient of variation (*n* = 3 measured at different days) based on the mean ionic intensity of the whole dataset (treated *n* = 4 and control *n* = 4), the accurate *m*/*z* value, and the putative chemical identification (ID). Most ions had a coefficient of variation (CV) under 35% and the average CV for the whole dataset (wdCV_avg_ ± SD) was 26 ± 12%. Of the 26 masses, 14 were also detected with MALDI-FT-ICR and 11 were putatively identified, of which seven species were identified as lipids (error < 1 ppm) (Table S1).

In order to evaluate the technical robustness of the method, the CV_avg_ ± SD was calculated for both intra- and inter-day measurements using multiple replicates for one of the biological xenograft models. There was more technical variation between measurements performed on different days (interday CV_avg_ = 33 ± 14%) compared to measurements from the same day (intraday CV_avg_ = 15 ± 9%).

### 2.2. Lipidomic Differences between Tumor and Necrotic Areas of Xenograft Tumors

In order to evaluate the spatial resolving ability of the method, the discriminating potential of the 26 ions was investigated comparing viable tumor and necrosis (obtained by histopathological annotations, Figure S1). This investigation was performed with univariate linear mixed model (LMM) testing and log_2_ fold change calculation (log_2_FC) using an adaption that accounts for the spatial autocorrection commonly observed in MSI data [17]. Of the 26 features tested, eight were significantly different (abs(log_2_FC) > 0.5, *p* < 0.05) between tumor and necrotic tissue in at least two technical replicates and are presented in Table 2. The full log_2_FC and LMM results for all 26 *m/z* values and for all three technical replicates are presented in Table S2.

**Figure 1:**
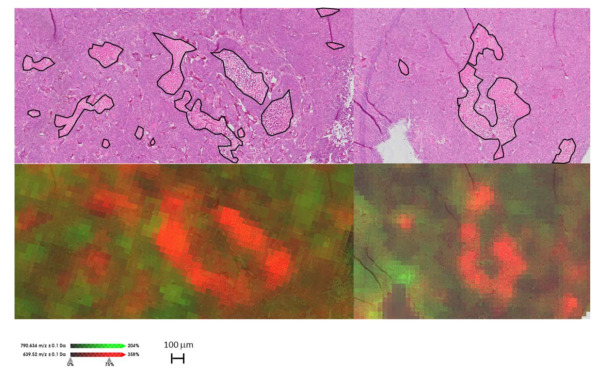
Example of MALDI-MSI ion maps compared with HES staining of the same tissue after MALDI MSI measurement. The distribution of the ions at *m/z* 790.6 (green) and 639.5 (red) distinguish tumor and necrotic regions, respectively. Necrotic areas are marked with black.

Of these eight *m/z* values, six had significantly higher intensities in tumor, while two were higher in the necrotic regions (*m/z* 639.5 and 683.5). The six masses (*m/z* 740.6, 766.6, 768.6, 788.6, 790.6, and 812.6) that were significantly higher in tumor, were reproducibly elevated in all three replicates and were identified as lipidic species (Table 1 and Table S2). Figure 1 shows the spatial distribution of two *m/z* values (*m/z* 639.5 and 790.6) that had high tumor-necrotic differentiation. It is worth noting that the same tissue used for MALDI MSI was HES stained afterward. Even after the MALDI process, it was still possible to identify tumor and necrotic areas from pre-processed sections compared with untouched sections, though morphology was somewhat compromised (Figure S2). This allowed for accurate co-registration with ionic images.

### 2.3. Differences Observed in the Tumor Lipidomes of Treated and Control Xenograft

**Figure 2:**
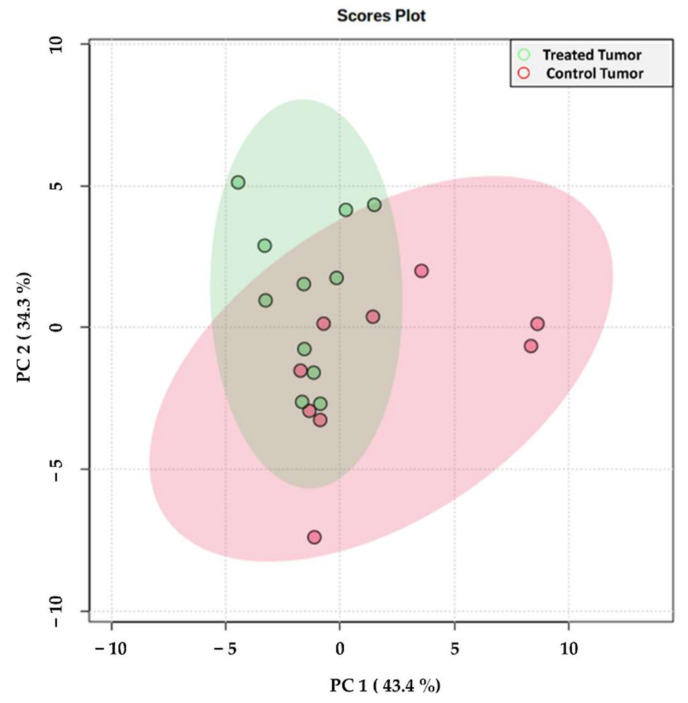
Principal component analysis score plot of the tumor regions from treated (green) and control (red) samples. Each spot represents an individual tissue section including all samples and their technical replicates (at least two for each sample). Colored circles represent 95% confidence intervals. Corresponding loading plot can be found in Figure S3.

In order to investigate the lipidomic alterations of the treated mice, principal component analysis (PCA) of the tumor regions (control and treated) was performed. As illustrated in the PCA score plot (Figure 2), the tumor regions of the treated mice showed separation from those of the control mice along the first and second components, which explained 43.4 and 34.3% of the variability within the dataset, respectively. The loading scores of those *m/z* features that had the greatest impact on this separation are presented as a loading plot in Figure S3. Interestingly, the tumors from the control samples also displayed a greater degree of variability among the different biological replicates, as indicated by the larger 95% confidence interval.

To then investigate those individual *m/z* features that had a significantly altered intensity among the treated and control tumors, and to support the findings of the PCA, pairwise comparisons were performed using the Wilcoxon Rank Sum Test, calculating log_2_FC, and the discriminatory power was assessed with receiver operative characteristic (ROC) analysis (Table 3).

**Figure 3:**
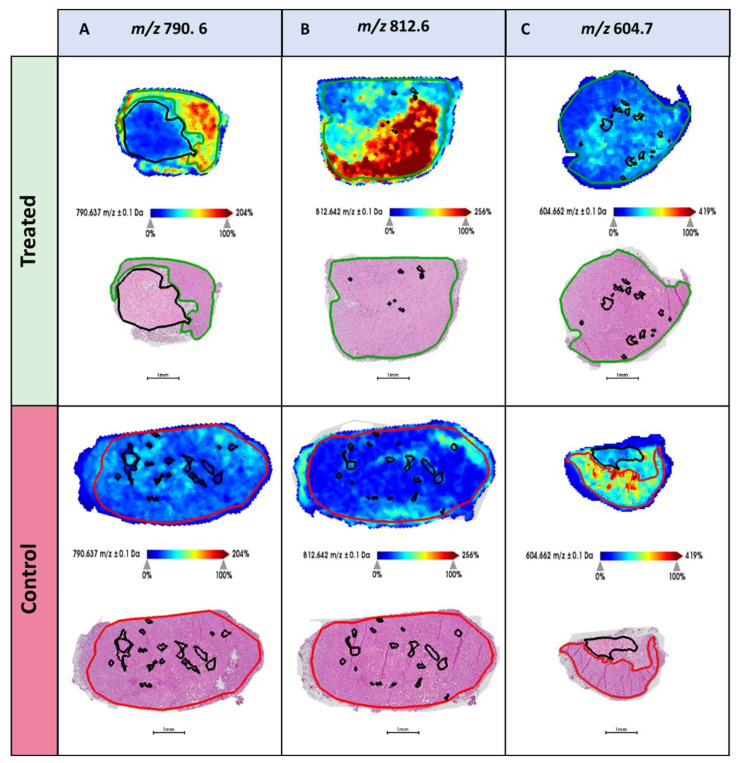
MALDI-MS images highlighting the tissue distribution of (**A**), *m/z* 790.6, (**B**) *m/z* 812.6, and (**C**) *m/z* 604.7 in (top) the treated and (bottom) control xenografts. HES stained images of each tissue section is provided below with the pathologist’s annotation of the tumor regions (green = treated tumor and red = control tumor). Necrotic regions are marked in black.

Table 3 highlights 6 *m/z* features that were statistically up- or down-represented between the treated and control tumors. Among them, *m/z* 768.6, 790.6, and 812.6 displayed significantly increased levels in the tumors of treated xenografts, and showed discriminatory power indicated by high AUC values. Conversely, *m/z* 604.7 was shown to have the greatest discriminatory power and displayed increased intensity in the tumors of control mice. Whilst a borderline significance was obtained for this feature (*p* = 0.06), it was, however, associated with the largest fold change of those reported. When comparing the distribution of these *m/z* features in the treated and control tissue, their differential intensity was observed in their respective tumor regions and is supportive of the aforementioned data. This is exemplified in the MALDI-MS single-ion images presented in Figure 3, showing the tissue localization of *m/z* 604.7, 790.6, and 812.6.

## 3. Discussion

Clinical and pre-clinical molecular analysis of cancer tissue is an important means of gaining both insights into tumor biology and identifying new potential biomarkers. However, the reliability of the results depends on precise, accurate, and reproducible quantitative analytical methods. This is particularly important when analyzing FFPE tissue with MALDI MSI. This method allows for metabolic analysis of biobanked tumor specimens with high translational value but requires multiple sample preparation steps and is prone to variation [18]. In this study, using a recently developed sample preparation protocol to analyze lipids from FFPE tissue [16], we not only demonstrate the robustness of the method, but also identify several lipid species that potentially hold value as cancer biomarkers.

A particular strength of our study is the validation of the robustness of the method using three technical replicates. Unfortunately, due to the high hands-on time typically required in MALDI MSI experiments, several technical replicates are rarely included to validate the results. Furthermore, sparsity of the clinical material may also hinder collecting several sections for technical validation. Still, validating results using technical replicates measured on different days is highly encouraged in mass spectrometry experiments [19]. It is perhaps extra important when working with MALDI MSI of FFPE tissue, which is particularly sensitive to day-to-day variations, due both to the necessary deparaffinization and antigen retrieval treatments, and to variation in matrix recrystallization during matrix application.

In our study, the technical reproducibility of the applied MALDI-MSI workflow was evaluated by the CV of the peak intensities and showed a wdCV_avg_ of 26 ± 11%. This is slightly above the recommended CV threshold of 20% stated by The European Medicine Agency (EMA) for analytical techniques used to determine the concentration of a given analyte in a biological matrix [20]. However, the method used in this study notably differs from the EMA requirements by its ability to determine a large number of compounds simultaneously, allowing profiling of the metabolic state rather than specific determination of a single compound. Furthermore, as MSI techniques, and MALDI in particular, tend to be more prone to variation, a wdCV_avg_ of 26 ± 11% shows that this method has a good technical reproducibility. Compared with previous reports, our protocol displays a high degree of robustness, as 26 ion species could be detected on different days. A previously reported lipidomic MALDI-MSI study of fresh-frozen bovine ovary tissue showed a CV <30% based also on three technical replicates, indicating that our protocol allows for the analysis of FFPE tissue with a similar performance to the less complex analysis of fresh-frozen tissue [21]. In addition, the intraday CV_avg_ (=15 ± 9%) of this study is comparable to intra-section variance CV_avg_ (=16 ± 7%) of 62 lipids obtained by the DESI-MSI of fresh-frozen esophageal adenocarcinoma biopsies [22]. The sample preparation for DESI-MSI does not require any matrix for the desorption and ionization of the analytes. Therefore, the intraday variation is not due to matrix application and is comparable to matrix-free based MSI technologies. On the other hand, the coefficient of variation increased when the subsequent sections were measured on different days ((interday CV_avg_ = 33 ± 14%). This may be explained by day-to-day technical variations due to differences in the performance of the mass spectrometer and sample preparation devices as well as changes in laboratory environmental conditions (e.g., temperature and humidity) [23]. Recently, batch effects, potential systematic sources of technical variation, were identified in MSI analysis of a large sample size by multivariate analysis of pixel-to-pixel, section-to-section, and slide-to-slide variations, suggesting existing approaches to mitigate the batch effects such as improved study design, quality controls, new normalization methods, and robust experimental workflows [24]. The importance of technical replicates in order to obtain a quantifiable variance and reproducibility should be a must for the development of any analytical method [25]. The fact that we could detect reproducible differences in analyte levels between tumor and necrotic tissue across all three replicates for six lipids (Table 2) highlights the robustness of both the sample preparation method and the metabolic findings of this study.

This study also shows a relative inter-instrumental robustness as most of the *m/z* values (14 of 26, Table 1) could be detected by high resolution mass spectrometry (FT-ICR) and seven ions were putatively identified with an error < 1 ppm (Table S2). Additionally, four *m/z* values could be identified with an increased error tolerance (<10 ppm). A less strict error tolerance of 10 ppm is not usually employed considering the outstanding mass resolving power of the FT-ICR mass spectrometer, but were applied in another study to identify peptides and glycans directly on tissue [26].

Not all *m/z* values detected with the MALDI-TOF MSI (rapifleX) could be found in the MALDI-FT-ICR MS (Solarix) measurements. The different detection ability between the MALDI-TOF and MALDI-FT-ICR MS may be due to differences in the MALDI source. The modulation of the laser beam profile and its spatial structure are critical factors for the performance of the MALDI process and therefore for the ionic intensity [27]. For example, the Solarix utilizes a structured Nd:YAG laser beam with a repetition rate of 2 kHz, whereas the rapifleX utilizes a smartbeam 3D laser with a repetition rate of 10 kHz. This has been suggested previously in the literature given that the comparison of peptide mass fingerprints for the same tryptic digested sample using MALDI-TOF and MALDI-FT-ICR showed differences in the total number of *m/z* signals detected, affecting the protein identification accuracy [28].

As presented in Table 1, five ions were identified as phosphatidylethanolamines (PE) and one ion as phosphatidylserine (PS). Both have a primary amino head group that is highly reactive with the formalin and, therefore, are the main lipid species incorporated into the cross-linking network during formalin fixation [29]. This chemical reaction extensively reduces the detection of PEs and PSs during the mass spectrometric analysis (LC-MS/MS and MSI) of formalin-fixed tissue [30,31]. Our results demonstrate that optimizing the sample preparation protocol with an antigen retrieval step can aid in the recovery of cross-linked lipids [16,31]. Moreover, despite PEs species being more easily detectable in negative mode [32], in this study, PEs were detected in positive mode as the [M + Na]^+^ adduct as previously observed by MALDI-MS [33]. The high sodium content of the buffered formalin solution used for tissue fixation potentially favor the detection of sodium adducts [34]. Finally, *m/z* 812.6 has previously been detected and identified as PS 36:1 [M + Na]^+^ by MALDI-MSI and LC-MS/MS in human renal cancer tissue [16], further confirming the findings of this study and the possible relevance of this lipid in the context of cancer biology. Our results successfully demonstrate reliably that including antigen retrieval is beneficial for recovering the PEs and PSs species from the cross-linked environments, extending the lipidomic analysis on FFPE tissues.

The method’s ability to discriminate between distinct biological compartments was demonstrated by comparing the lipid profiles of viable and necrotic tumor tissue (Table 2 and Figure 1). Necrotic foci appear when nutrient and oxygen deprivation causing cell death occurs and is often the result of a deteriorating microenvironment in rapidly growing tumors [35]. It has previously been shown that DESI-MSI can identify necrosis in fresh frozen tumor specimens [36] based on the presence of lipid species specific to viable and necrotic tissue, respectively. While fresh frozen tissue is a suitable material in the intra-operative setting, analysis of biobanked FFPE material requires methods optimized for the analysis of FFPE tissue. The necrosis-specific lipid profile identified in this study could potentially be used to determine the fraction of necrosis in heterogenous tumors in an automated manner, which could be of great value in translational cancer research. Biological interpretation of lipid chemistry in necrosis is challenging due to the combined effect of biologically regulated metabolism and the chemical degradation of lipid species in necrotic foci [37].

This study provides evidence of alterations in lipid metabolism in xenograft tumors following treatment with the glutaminase inhibitor CB-839. The *m/z* 812.6 (PS 36:1) and 790.6 (PE 38:4) are particularly interesting as these lipids had significantly higher levels in the treated tumors compared to the control tumors (Table 3 and Figure 3). The treated xenografts were administered with the glutaminase inhibitor CB-839, which inhibits the conversion of glutamine to glutamate. In previous studies, this drug has been shown to reduce the availability of glutamate both in responding and non-responding tumors [38]. While response to treatment has been associated with metabolic adaption to hypoxia, a global understanding of the biochemical consequences of glutaminase inhibition is still lacking. Several studies have pointed to a clinically relevant interplay between glutamine and lipid metabolism [39,40]. Notably, one of the lipids in our study with higher levels in the treated mice is a PE (*m/z* 790.6, PE 38:4). Glutamine availability has previously been shown to affect phospholipid synthesis, where glutamine starvation caused reduced levels of PEs, and glutamine supplement resulted in increased PE synthesis [41]. This could indicate that the glutaminase inhibitor causes elevated levels of glutamine (as it is no longer converted to glutamate), which in turn activates more PEs synthesis. The exact mechanism of how glutamine levels affect phospholipid synthesis is not entirely clear, and would benefit from more functional research.

We further observed notably less variation in the treated tumors compared to the controls (Figure 2). The glutaminase inhibitor could be killing specific cancer cell subgroups, leaving the treated tumors with less variation and tumor heterogeneity. Alternatively, the reduced growth rate of the treated tumors could similarly reduce the heterogeneity, which has previously been shown to be significant in the MAS98.06 xenografts [42]. As the effect of glutaminase inhibitors in clinical trials has been variable, methods for metabolic profiling of tumor tissue from test subjects could help identify predictive biomarkers or other traits that can contribute to discriminate between responding and non-responding patients.

To conclude, this study has demonstrated strong technical reproducibility of a newly developed protocol for lipid detection in FFPE tumor tissue using MALDI MSI. With this robust method we identified, for the first time, interesting metabolic lipid alterations between xenograft breast tumors treated with glutamine inhibitor compared to the control tumors in FFPE specimens. This included changed levels of specific lipids and reduced heterogeneity in the treated tumors. For future studies, it will be valuable to use this methodology to investigate the lipid profile of clinical archived breast cancer FFPE tissue, and its potential connection to treatment response, histopathological grade, and clinical outcome.

## 4. Materials and Methods

### 4.1. Chemicals

Unless indicated otherwise, all chemicals were purchased from Sigma-Aldrich (Oslo, Norway) and all the solvents were HPLC grade.

### 4.2. Biological Materials

The MAS98.06 patient-derived breast cancer xenograft was established at the Institute of Cancer Research, Oslo University Hospital, as previously described [43]. Tumor tissue was orthotopically implanted into 6-week old female Hsd:Athymic Nude-Foxn1nu mice. The animals were kept under pathogen-free conditions and received supplementary 17-β-estradiol (4 mg/mL) in the drinking water. When the tumors reached a size of 200–300 mm^3^, the mice received CB-839 (200 mg/kg) or placebo two times daily for 28 days, before tumors were harvested and snap-frozen in liquid nitrogen until further processing.

### 4.3. Sample Preparation and MALDI-MSI Analysis with Rapiflex

**Figure 4:**
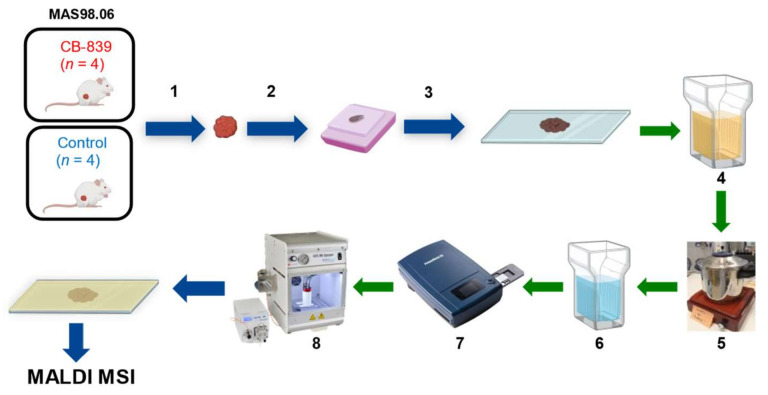
Sample preparation workflow for MALDI-MSI based lipidomics analysis on FFPE xenograft tissue. (1) Resection of the tumor, (2) fixation in formalin and embedding in paraffin block, (3) sectioning and mounting on conductive ITO slides, (4) solvent washing, (5) antigen retrieval, (6) hydration, (7) optical picture scanning, (8) matrix application by automatic sprayer.

The sample preparation workflow for MALDI-MSI is depicted in Figure 4.

The xenograft biopsies (*n* = 4 for treated and *n* = 4 for control) were, after being snap-frozen, formalin-fixed for 48 h, dehydrated and paraffin embedded. Sections of 4 µm thickness were mounted on ITO slides (MSI Diagnostics GmbH) in a random order and stored in a desiccator until analysis. The ITO slides were previously coated with poly-D-lysine. For each biological replicate, three sections were mounted as technical replicates. Separate FFPE sections were cut, mounted on glass slides, and stained with hematoxylin, erythrosine, and saffron (HES). The detailed staining protocol is described in the Supplementary Materials.

The sections were deparaffinized in toluol three times for 5 min and subsequently washed in isopropanol (5 min), EtOH 100% (5 min), EtOH 90% (5 min), and EtOH 70% (5 min). The sections were dried under a fume hood for 10 min. Antigen-retrieval was performed in a 10 mM citric acid solution (pH = 5.95) for 30 min at 120 °C using a pressure cooker. The sections were dried and then washed with LC/MS grade water (Fisher Chemical^TM^) prior to the matrix application for 2 min and scanned using a histology slide scanner (PrimeHisto XE Histology Slide Scanner) with an image resolution of 5000 dpi.

For the matrix solution, 100 mg of α-cyano-4-hydroxy-cinnamic acid (CHCA) was dissolved in 6.959 mL MeOH, sonicated for 3 min until CHCA was dissolved. A total of 48 microliters of aniline was added and then sonicated for 5 min in order to form the ionic liquid [44]. Afterward, 2.983 mL of LC/MS grade water was added, and finally 10 microliters of trifluoroacetic acid (TFA).

Matrix was applied on the tissue sections using the automatic HTX M5 Sprayer^TM^ (HTXImaging) with the following parameters: number passes 2, flow rate of 0.06 mL/min, velocity of 1200 mm/min, spray pattern CrissCross, and pressure of 5 psi as published previously [16]. The resulting matrix density was 1 × 10^−3^ mg/mm^2^.

MALDI MSI measurements were performed using a rapifleX MALDI Tissuetyper™ mass spectrometer (Bruker Daltonics, Bremen, Germany) equipped with a Smartbeam 3D laser under “Single” and with a digitizer frequency of 1.25 GHz. The analyzer was operated in positive reflector mode, and the laser was fired with a repetition rate of 10 kHz. External calibration was performed using red phosphorous dispersed in acetone, spotted beside the tissue section. The measurements were acquired in the *m/z* range 500–1300 using a lateral resolution of 50 μm and 250 laser shots pr. pixel. Further instrumental details are described in Table S3 of the Supplementary Materials.

### 4.4. Histopathological Assessment

All corresponding HES-stained sections were independently annotated by an experienced pathologist (A.M.B.) and areas of viable tumor, necrosis, and non-tumoral tissue (loose connective tissue, adipose tissue, benign glandular tissue) were delineated manually on the digital whole slide images (WSI). The spatial annotations were then transferred to the sections that had undergone MALDI-MSI measurements using SCiLS. For the purpose of robust statistical analysis, only the tissue areas that exclusively contained either tumor or necrotic tissue were selected, while areas with mixed tissue types were excluded. The tumor and necrotic specific areas used for the statistical analysis are depicted in Figure S4.

### 4.5. Data Analysis

The dataset was imported into SCiLS lab software (Version 2021c Pro, GmbH, Bremen, Germany) and normalized to the total ion count (TIC) of each individual spectrum. The mean spectra of each ROI (necrosis and tumor for each section) were exported from SCiLS and opened in the software mMass (Version 5.5.0) [45]. Here, the spectra were baseline corrected and peak picking were performed with S/N, absolute intensity, and relative intensity thresholds of 10.0, 0.14, and 0, respectively. Additionally, picking height was set to 100 and the ‘apply baseline’, ‘apply smoothing’, and ‘remove shoulder peaks’ choices were selected.

One aim of this study was to identify masses (*m/z* values) that are reproducible, both across all three technical replicates and the biological replicates. The strategy to achieve this is depicted in Figure S5. First, for each biological replicate, three lists of common masses were identified: tumor masses, necrosis masses, and masses with a consistent log_2_ fold change (log_2_FC) between the tumor and necrosis (in the same section). In short, masses detected in the mean tumor spectra across all three technical replicates were defined as common. To adjust for mass-shifts, *m/z*-values within a 220 ppm range of each other were defined as arising from the same mass. Furthermore, CV was calculated for each peak and peaks with a CV < 45% were kept. The same procedure was performed for mean necrotic spectra. For the log_2_FC peak list, masses that were detected in both tumor and necrotic regions in all technical replicates and had log_2_FC (comparing tumor and necrosis each section) with CV < 60% were kept. The three mass lists were then merged and duplicate *m/z*-values were removed. Next, in SCiLS, matrix peaks were identified and removed if they had higher levels outside the tissue and above 0.9 area under the curve (AUC) value when comparing on and off-tissue regions with receiver operating characteristic (ROC) analysis. The remaining masses of the peak list were visually inspected in SCiLS for the biological replicate sections, and masses that were off-tissue, isotopes, or had a low-quality signal were removed. This was performed for each biological replicate, resulting in eight different peak lists. Masses that were present in at least two biological replicates and that had a mean intensity > 1 (TIC normalized) were selected for the master peak list. A last quality check removed additional masses that were matrix-related and isotopes from the master peak list.

To investigate differences between tumor and necrotic regions, univariate linear mixed model (LMM) testing was performed for each technical replicate separately, using biological replicate as the random effect. LMM was performed in R (version 4.0.3) using the *nlme* (v3.1-152) package (Pinheiro J, Bates D, DebRoy S, Sarkar D, R Core Team (2021). nlme: Linear and Nonlinear Mixed Effects Models. R package version 3.1-152, https://CRAN.R-project.org/package=nlme (accessed on 5 June 2021)). An iterative fraction-based approach of LMM was used [46,47]. In short, LMM testing was applied using 1% randomly selected single spectra and was repeated 100 times. This strategy aims to account for the spatial autocorrection commonly observed in MSI data [17] and is described in more detail by Andersen et al. [46]. The reported log_2_FC and Benjamini–Hochberg adjusted *p*-values are the average values across all 100 iterations. Features with abs(log_2_FC) > 0.5 and *p* < 0.05 were considered significantly different. For technical replicate 2, three of the sections were excluded from the LMM analysis due to paraffin contamination, of which one was treated (CB2) and two were control (Ctr3 and Ctr5) samples. In addition to comparing tumor to necrosis for all biological replicates, LMM was also used to investigate the tumor compared to necrosis in the treated and control samples separately.

For the technical reproducibility evaluation, three types of CVs were calculated: one for the whole dataset in addition to inter- and intraday measurements CVs. All CV calculations were based on the mean ion intensities for the *m/z*-values of the master peak list. The whole dataset CVs were calculated using the average mass spectra of all tissue sections (including all technical replicates). Afterward, the average of the CV and standard deviation (SD) per sample were calculated, resulting in the whole dataset average CV (wdCV_avg_). The intraday and interday CV_avg_ were calculated in the same manner, but were based on three technical replicates measured on the same day and on different days, respectively.

To investigate the lipidomic alterations in the tumors of mice administered with the glutaminase inhibitor CB-839, unsupervised principal component analysis (PCA) was performed as well as pairwise comparisons using the Wilcoxon Rank Sum Test and receiver operating characteristic (ROC) analysis. The Wilcoxon Rank Sum Test, fold change, and principal component analysis (PCA) was performed on the *m/z* intensities of the mean spectra using MetaboAnalyst 5.0 [48]. An *m/z* feature was considered to be significant if a *p*-value < 0.05 was obtained for at least two out of three technical replicates of a biological replicate. Meanwhile, ROC analysis was performed in SCiLS Lab (Version 2021c Pro, GmbH, Bremen, Germany) on equally sized subsets of treated and control tumor spectra (*n* = 15,000). An *m/z* feature was considered significant when an AUC value was ≥0.7 in at least two out of three technical replicates of a biological replicate, and at least three out of four biological replicates. The reported AUC value represents the mean value obtained from each of the technical replicates that met the above criteria.

### 4.6. Lipids Identification by MALDI FT-ICR

Additional MALDI-MS and MALDI imaging experiments were performed in order to aid in lipid identification using a 12T Bruker FTICR mass spectrometer (Bruker Daltonics, Bremen, Germany) equipped with a dual MALDI/ESI ion source. All imaging experiments were performed using a spatial resolution of 50 µm and the minimum laser spot size. A total of 20 laser shots were used per pixel. Data were collected in positive ionization mode in the mass range *m/z* 150–2000. External calibration was performed prior to analysis using the electrospray ion source and NaTFA clusters. FlexImaging (Bruker Daltonics,) was used to visualize the imaging data and DataAnalysis was used to visualize individual mass spectra. Putative identification of the ions was performed by accurate mass determination and database search in the human metabolome database (HMDB) [49] and LIPID MAPS with a tolerance threshold ≤10 ppm. The chemical assignment was performed by prioritizing the compounds observed experimentally than the ones theoretically computed.

## Figures and Tables

**Table 1:** Master peak list with *m/z* values, corresponding coefficients of variation (CV), accurate *m/z* values and chemical identification (ID). CV was calculated for the three technical replicates of the whole dataset (treated *n* = 4 and control *n* = 4).

*m/z*	CV (%)	Accurate *m/z*	ID [Adduct]^+^
534.6	41		n.i. ^(1)^
548.6	50		n.i. ^(1)^
562.6	45		n.i. ^(1)^
576.7	9		n.i. ^(1)^
604.7	50		n.i. ^(1)^
609.4	14	609.340	PI 20:1 [M + H − H_2_O]^+ (2,4)^
639.5	32	639.408	PA 32:5 [M+H]^+ (3,4)^
683.5	22	683.434	PG 31:4 [M + H − H_2_O]^+ (3,4)^
707.6	23		n.i. ^(1)^
722.5	21		n.i. ^(1)^
727.6	16	727.460	PG 33:5 [M + H]^+ (3,4)^
740.6	31	740.520	PE 34:1 [M + Na]^+ (2)^
758.6	23		n.i. ^(1)^
766.6	29	766.536	PE 36:2 [M + Na]^+ (2)^
768.6	33	768.554	PE 38:4 [M + H]^+ (2)^
771.6	8	771.486	PA 40:6 [M + Na]^+ (3,4)^
779.3	18	779.254	n.i.
784.6	23		n.i. ^(1)^
786.6	23		n.i. ^(1)^
788.6	28	788.5201	PE 38:5 [M + Na]^+ (2)^
790.6	32	790.536	PE 38:4 [M + Na]^+ (2)^
801.2	16	801.193	n.i.
812.6	32	812.541	PS (36:1) [M + Na]^+ (2)^
815.6	14		n.i. ^(1)^
826.6	8		n.i. ^(1)^
867.2	30	867.088	n.i.

n.i. not identified; ^(1)^ not detected in MALDI FT-ICR; ^(2)^ Identified with an error < 1 ppm; ^(3)^ Identified with an error between 8 and 10 ppm; ^(4)^ not observed experimentally.

**Table 2:** Fold change and univariate linear mixed models testing comparing tumor and necrosis. Log_2_ fold change (Log_2_FC) and *p*-values are the mean values across all three technical replicates. Masses that were significant (abs(log_2_FC) > 0.5, *p* < 0.05) in at least two replicates are presented. Positive log_2_FC indicates increase in tumor compared to necrosis.

Tumor vs. Necrosis		
*m/z*	Log_2_FC (Range)	*p*-Value (Range)
639.5	−0.61 (−0.8, −0.38)	0.018 (0.001, 0.041)
683.5	−0.62 (−0.67, −0.59)	0.034 (0.023, 0.05)
740.6	0.68 (0.5, 0.84)	0.011 (0.002, 0.028)
766.6	0.89 (0.81, 1.02)	0.01 (0.001, 0.026)
768.6	0.87 (0.8, 0.9)	0.02 (0.004, 0.044)
788.6	0.8 (0.67, 1.07)	0.015 (0.002, 0.037)
790.6	1.01 (0.85, 1.17)	0.011 (0, 0.033)
812.6	1.03 (0.72, 1.39)	0.015 (0.001, 0.024)

**Table 3:** Summary of the fold change (log_2_FC), *p*-value determined by the Wilcoxon Rank Sum Test, the principal component (PC) loading scores, and AUC values when comparing tumors of the treated and control xenografts.

Control Tumor vs. Treated Tumor					
*m/z*	log_2_FC	*p*-Value	AUC	Group	
768.6	−0.34	0.01	0.71	**↑**	Treated Tumor
784.6	−0.23	0.08	0.70	**↑**	Treated Tumor
786.6	−0.19	0.08	0.75	**↑**	Treated Tumor
790.6	−0.30	0.03	0.70	↑	Treated Tumor
812.6	−0.42	0.03	0.70	**↑**	Treated Tumor
604.7	1.11	0.06	0.87	↑	Control Tumor
779.3	0.53	0.01	0.65	**↑**	Control Tumor
801.2	0.50	0.01	0.64	**↑**	Control Tumor
867.2	0.58	0.02	0.63	**↑**	Control Tumor

## Data Availability

The data that support the findings of this study are available from the corresponding author, M.G., upon reasonable request.

## Supplementary Materials

The following are available online at https://www.mdpi.com/article/10.3390/metabo11090577/s1. HES-Staining protocol, Figure S1: Histopathological annotations of patient derived breast cancer xenografts, Figure S2: HES-stained sections from patient-derivative xenograft breast cancer tissue, Figure S3: Principal component analysis loading plot, Figure S4: Tissue areas considered for statistical analysis of MSI experiments, Figure S5: Overview of peak selection process, Table S1: Putative chemical identification by accurate mass determination performed by high resolution mass spectrometry (MALDI-FT-ICR), Table S2: Fold change and univariate linear mixed models testing comparing tumor and necrosis in technical replicate 1, 2 and 3, Table S3: Instrumental parameters employed for MALDI-MSI (rapifleX).
