# Supplementary material for: Reproducible Lipid Alterations in Patient-Derived Breast Cancer Xenograft FFPE Tissue Identified with MALDI MSI for Pre-Clinical and Clinical Application
Source: Metabolites. 2021 Aug 26;11(9):577. doi: 10.3390/metabo11090577 (PMC8467053; doi:10.3390/metabo11090577)
Supplement: Supplementary file 1 [file metabolites-11-00577-s001.zip › metabolites-1331883-supplementary.pdf]

metabolites

Reproducible Lipid Alterations in Patient-Derived Breast Cancer Xenograft FFPE Tissue Identified with MALDI MSI for Pre-Clinical and Clinical Application

Vanna Denti [1,†], Maria K. Andersen [2,†], Andrew Smith [1], Anna Mary Bofin [3], Anna Nordborg [4], Fulvio Magni [1], Siver Andreas Moestue [3,5] and Marco Giampà [3,*]

1 Proteomics and Metabolomics Unit, Department of Medicine and Surgery, University of Milano-Bicocca, 20854 Vedano al Lambro, MB, Italy; v.denti@campus.unimib.it (V.D.); andrew.smith@unimib.it (A.S.); fulvio.magni@unimib.it (F.M.)
2 Department of Circulation and Medical Imaging, NTNU—Norwegian University of Science and Technology, 7491 Trondheim, Norway; maria.k.andersen@ntnu.no
3 Department of Clinical and Molecular Medicine, NTNU—Norwegian University of Science and Technology, 7491 Trondheim, Norway; anna.bofin@ntnu.no (A.M.B.); siver.a.moestue@ntnu.no (S.A.M.)
4 Department of Biotechnology and Nanomedicine, SINTEF, 7034 Trondheim, Norway; anna.nordborg@sintef.no
5 Department of Pharmacy, Nord University, 8026 Bodø, Norway
* Correspondence: marco.giampa@ntnu.no
† These authors contributed equally to this work.

HES-staining protocol

The staining of formalin fixed, paraffin embedded tissue sections was performed using automatic slide stainer, (Tissue-Tek © Prisma™, Sakura)

1. Heat sections at 60 degrees for 10 minutes.
2. Deparaffinize sections in Tissue Clear to remove the paraffine, 3 x 3 min.
3. Rehydrate in graded ethanols to water: ethanol 100% x 3, ethanol 96%, ethanol 80% and water, each 1 min.
4. Wash in water, 1 min.
5. Stain in Haematoxylin, 5 min.
6. Rinse in running water, 8 min.
7. Stain in Erythrosin, 5 min.
8. Rinse in running water, 1 min
9. Dehydrate in graded ethanols (80%, 96% - 100% ethanol), each 1 min.
10. Stain in Saffron, 5 min.
11. Rinse in ethanol 100 %, 3 x 1 min.
12. Tissue Clear, 3 x 1 min.
13. Coverslipping with TissueMount in Tissue-Tek©Glas™ coverslipper (Sakura).

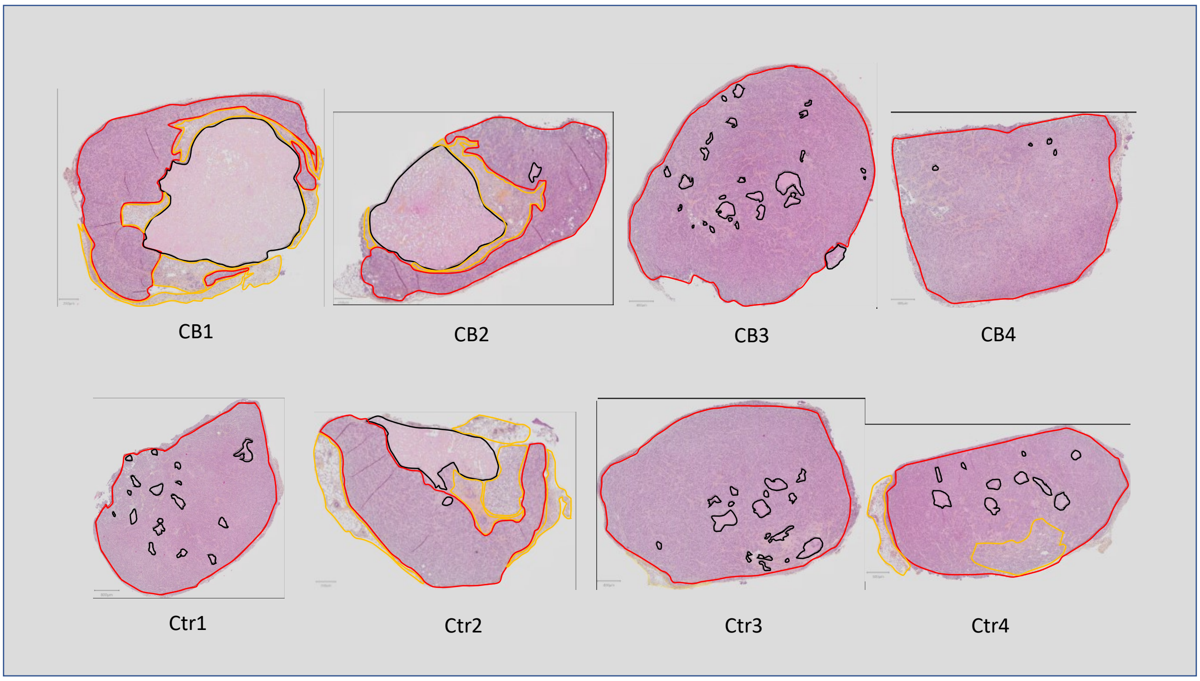

Figure S1. Histopathological annotations of patient derived breast cancer xenografts. CB 1-4 and Ctr 1-4 are xenograft treated with CB-839 and control, respectively. The tumor, necrotic and non-tumoral (loose connective tissue, adipose tissue, benign glandular tissue) regions are annotated with red, black and orange lines, respectively.

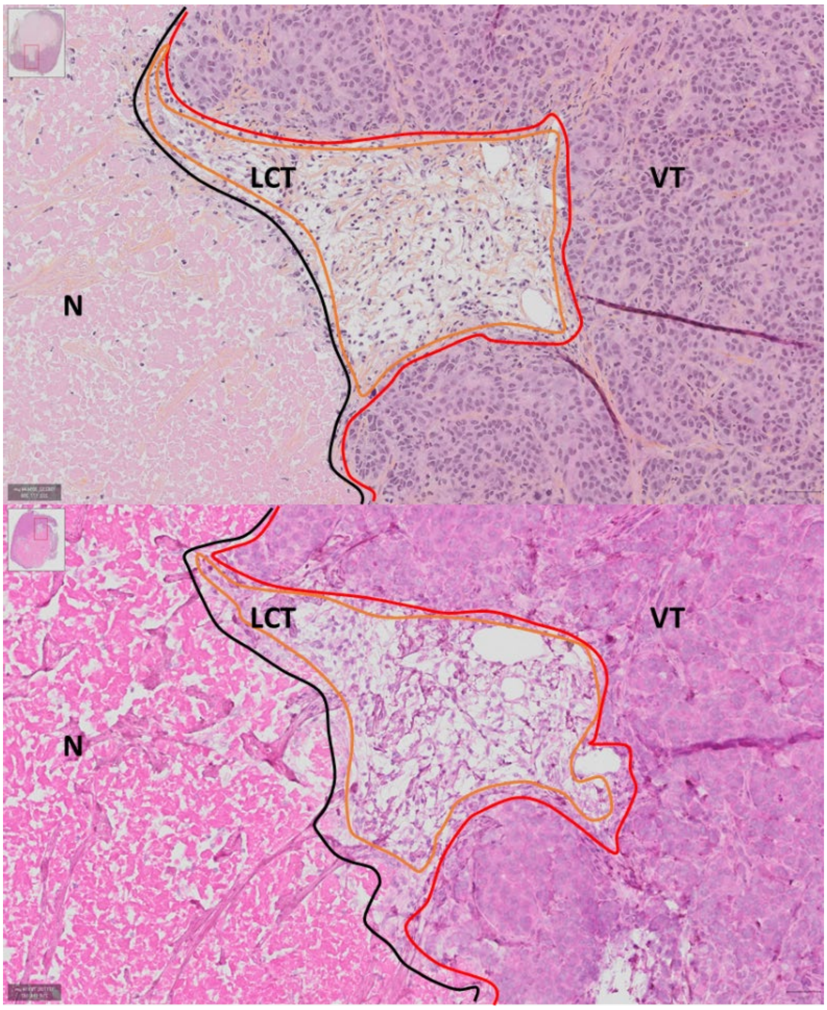

Figure S2. HES-stained sections from patient-derivative xenograft breast cancer tissue: top) baseline HES-stained tissue section, and bottom) Section stained with HES after antigen retrieval and the MALDI procedure. While the histological integrety is somewhat compromised in the bottom image, it is still possible to identify the three areas of interest in the section,viable tumour (VT) necrotic tumor tissue (N) non-tumor tissue (LCT) after antigen retrieval and MALDI MSI.

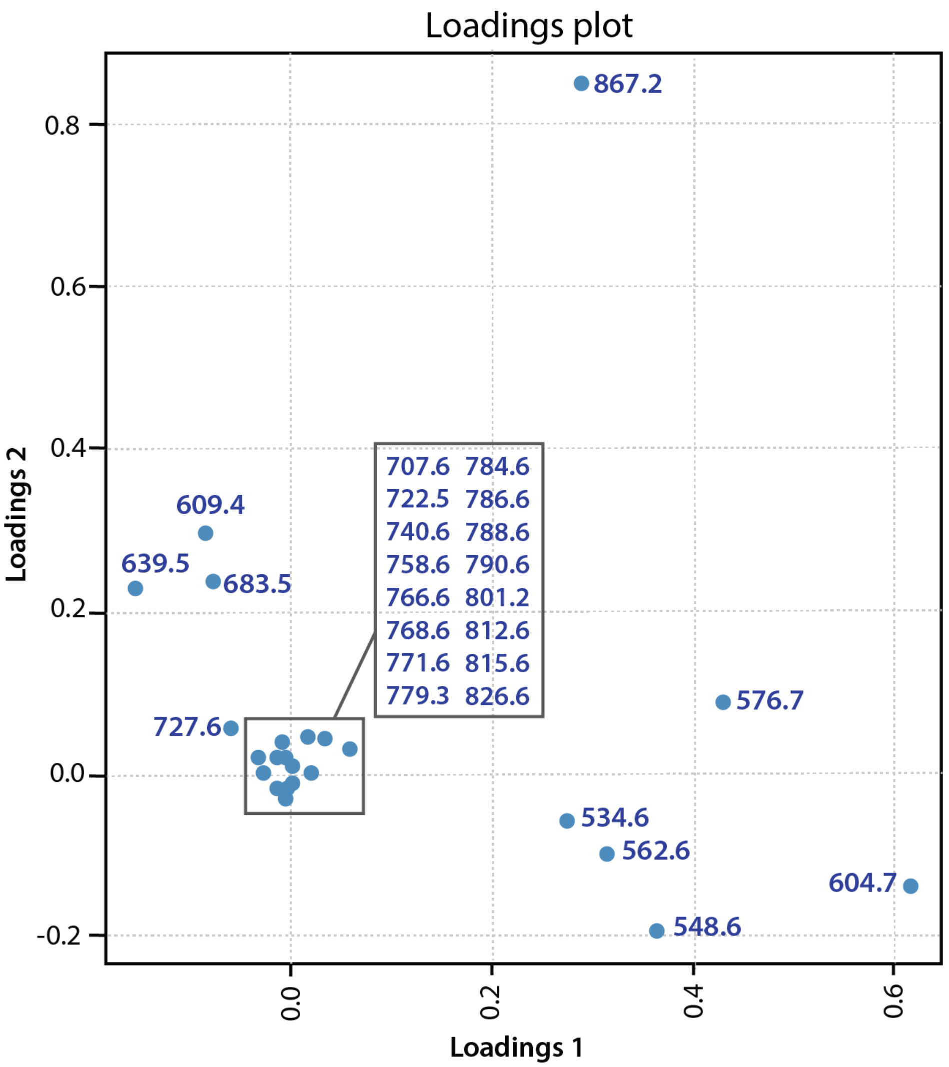

Figure S3. Principal component analysis loading plot. The loading plot corresponds to the scores plot in Figure 2 and the data point labels are *m*/*z*-values.

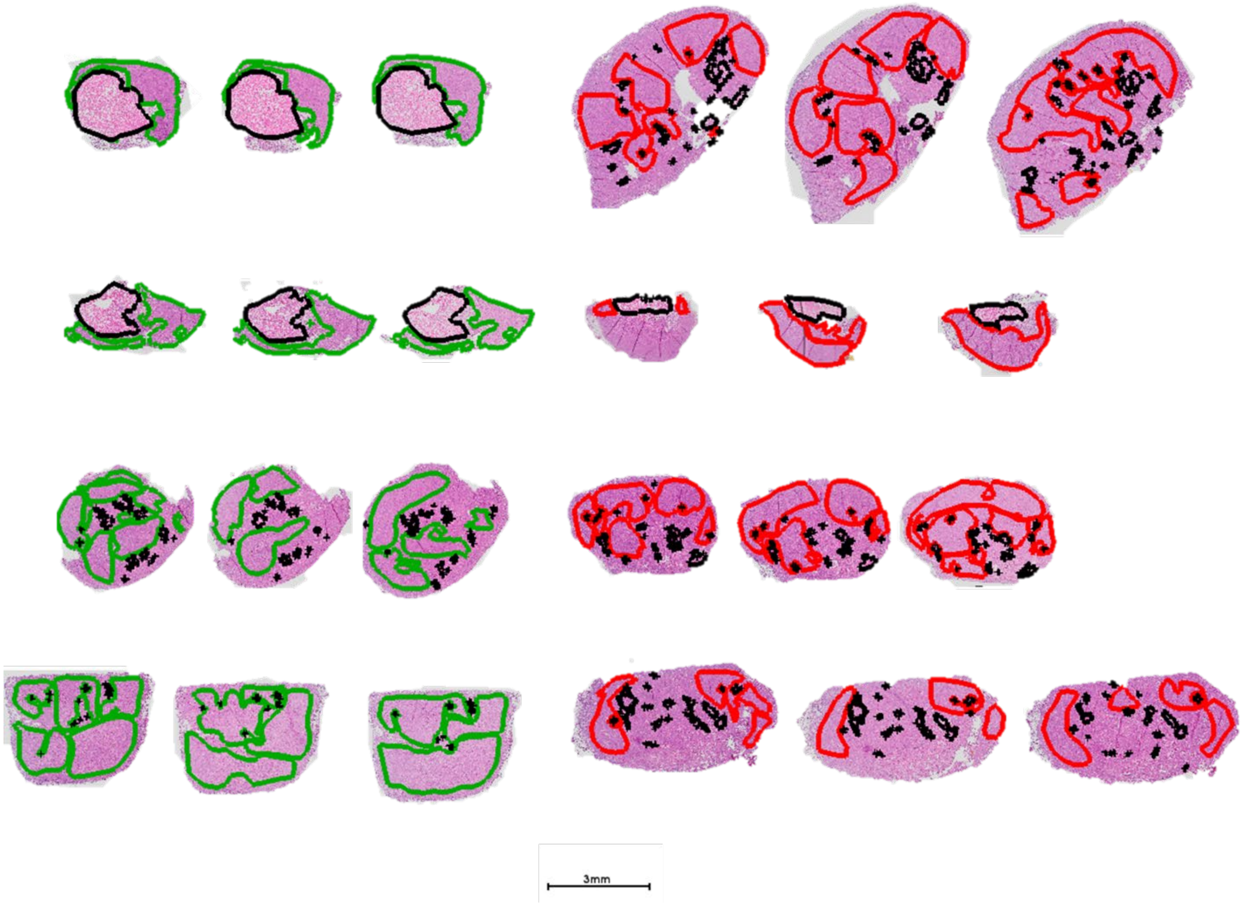

Figure S4. Tissue areas considered for statistical analysis of MSI experiments. Control and treated viable tumor areas are deliniat-ted with red and green, respectively. Necrotic areas are deliliniated with black.

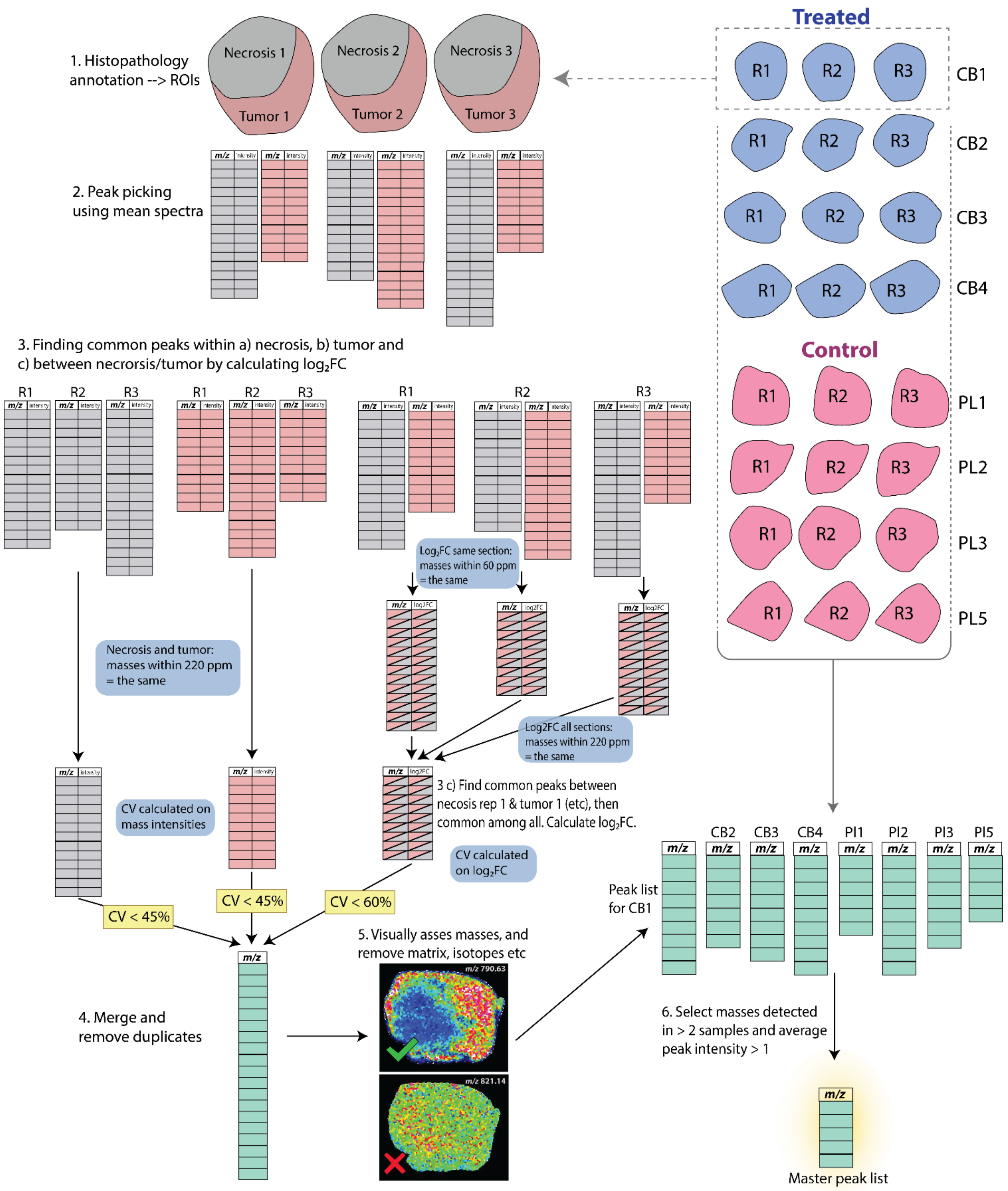

Figure S5. Overview of peak selection process. For each biological sample, 1. regions of interest (ROI) were drawn based on histopathology annotation of tumor and necrotic areas. 2. Peak picking was performed on the mean spectra for each ROI. 3. Three lists of common masses were identified: tumor masses, necrotic masses and masses with a consistent log_2 fold change (log_2FC) between tumor and necrosis. 3 a) Masses detected in the mean tumor spectra across all three technical replicates were defined as common. To adjust for mass-shifts, *m*/*z*-values within a 220 ppm range were defined as arising from the same mass. Further, CV was calculated for each peak and peaks with a CV < 45% were kept. 3 b) The same procedure was performed for mean necrotic spectra. 3 c) For the log_2FC peak list, masses that were detected in both tumor and necrotic regions in all technical replicates and had log_2FC (comparing tumor and necrosis each section) with CV < 60% were kept. 4.

The three mass lists were then merged and duplicate *m/z*- values were removed. 5. Next, the masses of the peak list were visually inspected in SCiLS for the biological replicate sections, and masses that were off-tissue, isotopes or had a low-quality signal were removed. This was performed for each biological replicate resulting in eight different peak lists. 6. Masses that were present in at least two biological samples and that had a mean intensity > 1 (TIC normalized) were selected for the master peak list. Note that sample shape, ROI and peak list lengths are illustrative only and does not represent the true shape or length of the samples or peak lists.

Table S1. Putative chemical identification by accurate mass determination performed by high resolution mass spectrometry (MALDI-FT-ICR)

m/z TOF	Experimental *m/z*	Theoretical *m/z*	Chemical formula	Error (ppm)	Assigment
609.4	609.340	609.3404	$[C_{29}H_{55}O_{12}P + H - H_2O]^+$	0.7	PI 20:1
639.5	639.408	639.4020	$[C_{35}H_{59}O_8P + H]$	9.4	PA 32:5
683.5	683.434	683.4282	$[C_{37}H_{65}O_{10}P + H - H_2O]$	8.5	PG 31:4
727.6	727.460	727.4545	$[C_{39}H_{67}O_{10}P + H]^+$	7.6	PG 33:5
740.6	740.520	740.5201	$[C_{39}H_{76}NO_8P + Na]^+$	0.1	PE 34:1
766.6	766.536	766.5357	$[C_{41}H_{78}NO_8P + Na]^+$	-0.4	PE 36:2
768.6	768.554	768.5538	$[C_{43}H_{78}NO_8P + H]^+$	-0.3	PE 38:4
771.6	771.486	771.4935	$[C_{43}H_{73}O_8P + Na]$	-9.7	PA 40:6
779.2	779.254				n.i
788.6	788.520	788.5201	$[C_{43}H_{76}NO_8P + Na]^+$	0.1	PE 38:5
790.6	790.536	790.5357	$[C_{43}H_{78}NO_8P + Na]^+$	-0.4	PE 38:4
801.2	801.193				n.i.
812.6	812.541	812.5412	$[C_{42}H_{80}NO_{10}P + Na]^+$	0.2	PS 36:1
867.1	867.088				n.i.

n.d. not identified

Table S2. Fold change and univariate linear mixed models testing comparing tumor and necrosis in technical replicate 1, 2 and 3. Masses with absolute log_2 fold change (log_2FC) > 0.5 and p-value < 0.05 are considered significant and are marked in bold. All p-values are Benjamini-Hochberg adjusted.

m/z	Replicate 1		Replicate 2		Replicate 3	
	Log_2FC	p-value	Log_2FC	p-value	Log_2FC	p-value
534.6	-0.24	0.190	0.30	0.450	0.33	0.300
548.6	0.13	0.560	0.42	0.590	0.32	0.470
562.6	-0.28	0.130	0.22	0.490	0.11	0.520
576.7	-0.18	0.110	0.25	0.450	0.16	0.370
604.7	0.14	0.390	0.65	0.380	0.47	0.250
609.4	-0.16	0.100	-0.57	0.005	-0.69	0.000
**639.5**	**-0.65**	**0.012**	**-0.38**	**0.041**	**-0.80**	**0.001**
**683.5**	**-0.60**	**0.023**	**-0.59**	**0.050**	**-0.67**	**0.030**
707.6	0.25	0.210	-0.15	0.300	-0.03	0.480
722.5	0.06	0.430	-0.08	0.370	-0.04	0.430
727.6	-0.47	0.039	-0.31	0.058	-0.63	0.018
**740.6**	**0.70**	**0.002**	**0.50**	**0.028**	**0.84**	**0.002**
758.6	0.23	0.260	0.38	0.200	0.10	0.530
**766.6**	**0.85**	**0.001**	**0.81**	**0.026**	**1.02**	**0.002**
**768.6**	**0.90**	**0.012**	**0.80**	**0.044**	**0.89**	**0.004**
771.6	-0.35	0.080	-0.38	0.058	-0.40	0.060
779.3	0.22	0.150	-0.10	0.530	0.02	0.500
784.6	0.63	0.012	0.65	0.120	0.73	0.011
786.6	0.60	0.003	0.52	0.082	0.52	0.009
**788.6**	**0.67**	**0.005**	**0.67**	**0.037**	**1.07**	**0.002**
**790.6**	**1.17**	**0.000**	**0.85**	**0.033**	**1.02**	**0.001**
801.2	0.17	0.400	-0.25	0.420	-0.16	0.280
**812.6**	**1.39**	**0.001**	**0.72**	**0.024**	**0.98**	**0.020**
815.6	0.04	0.460	-0.10	0.310	-0.08	0.370
826.6	-0.09	0.054	0.10	0.600	0.20	0.240
867.2	0.66	0.038	-0.40	0.310	-0.25	0.240

Table S3. Instrumental parameters employed for MALDI-MSI (rapifleX)

Parameter	Parameter value
Mode	Reflectron
Polarity	Positive
IonSource1	20.000 kV
PIE Voltage	2.610 kV
Lens	11.600 kV
Reflector	20.840 kV
Reflector2	1.085 kV
Reflector3	8.7 kV
PieDelay	120 ns
DetectorGain	2345 V
MassRange	*m/z* 500-1300
DeflectionConstant	0.326519
CutOffMass	399.975
Laser Shots	250
PowerUV	35
Frequency	10 kHz
